# Complete Genome Sequence of *Streptomyces venezuelae* ATCC 15439, Producer of the Methymycin/Pikromycin Family of Macrolide Antibiotics, Using PacBio Technology

**DOI:** 10.1128/genomeA.00337-16

**Published:** 2016-05-05

**Authors:** Jingxuan He, Anitha Sundararajan, Nicholas P. Devitt, Faye D. Schilkey, Thiruvarangan Ramaraj, Charles E. Melançon

**Affiliations:** a Department of Chemistry and Chemical Biology, University of New Mexico, Albuquerque, New Mexico, USA; b National Center for Genome Resources (NCGR), Santa Fe, New Mexico, USA; c Department of Biology, University of New Mexico, Albuquerque, New Mexico, USA; d Center for Biomedical Engineering, University of New Mexico, Albuquerque, New Mexico, USA

## Abstract

Here, we report the complete genome sequence of *Streptomyces venezuelae* ATCC 15439, a producer of the methymycin/pikromycin family of macrolide antibiotics and a model host for natural product studies, obtained exclusively using PacBio sequencing technology. The 9.03-Mbp genome harbors 8,775 genes and 11 polyketide and nonribosomal peptide natural product gene clusters.

## GENOME ANNOUNCEMENT

Actinobacteria are well known for their ability to produce a variety of structurally complex, often bioactive natural products that are useful as drugs, drug leads, and chemical probes (1, 2). Among Actinobacteria, members of the genus *Streptomyces* are some of the most frequently observed species in nature, with >575 validly published species names (3) and >18,000 publicly deposited 16S rRNA gene sequences reported to date. Since 2001, when the first *Streptomyces* genomes, those of the model organisms *S. coelicolor* A3(2) (4) and *S. avermitilis* MA-4680 (5), were sequenced, the genomes of nearly 700 *Streptomyces* strains have been sequenced to at least the draft stage and made publicly available. However, among these, only 35% (245 genomes) are high-quality (<100 scaffold) assemblies, and only 8% (56 genomes) are complete genomes.

The full complement of genes required for the biosynthesis of bacterial natural products are almost invariably found at specific genomic loci called natural product biosynthetic gene clusters (BGCs) (6), which typically range in size from ~10 kb to >100 kb. A sufficiently high-quality (typically <100 scaffolds) genome assembly is an important prerequisite for obtaining the intact natural product BGC sequences needed for accurate bioinformatics-guided natural product discovery (7, 8) and synthetic biology-based natural product production (9) efforts.

There is an extensive collection of molecular genetic tools available for use in *Streptomyces*, and several model *Streptomyces* hosts, including *S. coelicolor* A3(2) (4, 10), *S. lividans* strains, *S. avermitilis* MA-4680 (5, 11), *S. albus* J1074 (12), *S. venezuelae* ATCC 10712 (13), and *S. venezuelae* ATCC 15439, have been developed. *S. venezuelae* ATCC 15439, a producer of the methymycin/pikromycin family of macrolide antibiotics (14), has been a model host for studying and manipulating deoxysugar and polyketide biosynthesis and macrolide glycosylation (15), for heterologous production of natural products (16), and recently for unnatural amino acid incorporation (17). *S. venezuelae* ATCC 15439 is an advantageous model host because it is among the fastest growing *Streptomyces* strains (doubling time, ~60 min) (16), it grows in a dispersed manner in liquid culture, and it can be transformed efficiently. The complete nearly error-free genomes of model *Streptomyces* strains have been invaluable guides in the effort to understand and manipulate secondary metabolism.

To extend the capabilities afforded by a high-quality genome sequence to the model host *S. venezuelae* ATCC 15439, we sequenced its genome using PacBio next-generation technology. Genome sequencing was carried out using the Pacific Biosciences RSII (Menlo Park, CA) sequencing platform. PacBio long reads (two single-molecule real-time [SMRT] cells, ≈80× coverage) were assembled using the Hierarchical Genome Assembly Process 2 (HGAP2) protocol from SMRT Analysis version 2.0 package (18), resulting in the complete linear 9,034,396-bp *S. venezuelae* ATCC 15439 genome.

Gene prediction and annotation were carried out using RAST (19), incorporating the Glimmer (20) algorithm, and identified 8,682 putative protein-coding genes, 7 rRNA operons, and 72 tRNAs. Eleven polyketide and nonribosomal peptide natural product biosynthetic gene clusters, including the nearly error-free pikromycin cluster, were identified using *Dynamite* (8) and confirmed using antiSMASH (21). The *S. venezuelae* ATCC 15439 genome sequence will be a valuable resource for the continued development of the strain as a model host for natural product biosynthesis and synthetic biology studies.

### Nucleotide sequence accession numbers.

This genome sequence was deposited in EMBL/GenBank under accession no. LN881739. The version described in this paper is the first version, LN881739.1.
